# Shifts in temperature influence how *Batrachochytrium dendrobatidis* infects amphibian larvae

**DOI:** 10.1371/journal.pone.0222237

**Published:** 2019-09-19

**Authors:** Paul W. Bradley, Michael D. Brawner, Thomas R. Raffel, Jason R. Rohr, Deanna H. Olson, Andrew R. Blaustein

**Affiliations:** 1 Environmental Sciences Graduate Program, Oregon State University, Corvallis, Oregon, United States of America; 2 Department of Integrative Biology, Oregon State University, Corvallis, OR, United States of America; 3 Department of Biology, Oakland University, Rochester, MI, United States of America; 4 Department of Integrative Biology, University of South Florida, Tampa, FL, United States of America; 5 USDA Forest Service, Pacific Northwest Research Station, Corvallis, OR, United States of America; University of South Dakota, UNITED STATES

## Abstract

Many climate change models predict increases in frequency and magnitude of temperature fluctuations that might impact how ectotherms are affected by disease. Shifts in temperature might especially affect amphibians, a group with populations that have been challenged by several pathogens. Because amphibian hosts invest more in immunity at warmer than cooler temperatures and parasites might acclimate to temperature shifts faster than hosts (creating lags in optimal host immunity), researchers have hypothesized that a temperature shift from cold-to-warm might result in increased amphibian sensitivity to pathogens, whereas a shift from warm-to-cold might result in decreased sensitivity. Support for components of this climate-variability based hypothesis have been provided by prior studies of the fungus *Batrachochytrium dendrobatidis* (Bd) that causes the disease chytridiomycosis in amphibians. We experimentally tested whether temperature shifts before exposure to *Batrachochytrium dendrobatidis* (Bd) alters susceptibility to the disease chytridiomycosis in the larval stage of two amphibian species–western toads (*Anaxyrus boreas*) and northern red legged frogs (*Rana aurora*). Both host species harbored elevated Bd infection intensities under constant cold (15° C) temperature in comparison to constant warm (20° C) temperature. Additionally, both species experienced an increase in Bd infection abundance after shifted from 15° C to 20° C, compared to a constant 20° C but they experienced a decrease in Bd after shifted from 20° C to 15° C, compared to a constant 15° C. These results are in contrast to prior studies of adult amphibians highlighting the potential for species and stage differences in the temperature-dependence of chytridiomycosis.

## Introduction

Climate change represents one of the greatest challenges to biodiversity and conservation because it might compromise ecosystem functions worldwide. Most studies of climate-change induced effects on ecological communities emphasize the role of predicted changes to annual or seasonal mean temperature or precipitation metrics [1, 2]. However, many climate change models predict increases in the frequency and magnitude of extreme weather events, such as heat waves and droughts [3, 4], that can lead to increases in temperature variability at monthly to weekly timescales [5, 6]. These predicted climate-change induced increases in short-term temperature fluctuations could affect species interactions [7–9]. Yet few studies have investigated how increases in temperature variability affect disease dynamics despite the likelihood that such variability might differentially affect hosts and pathogens [10–12]. Ectotherms, such as amphibians, are particularly sensitive to climate change [13–16] and are experiencing disease-associated population declines and extinctions worldwide [17–20], making them an ideal group to investigate the relationship between temperature shifts and disease risk.

The aquatic chytrid fungal pathogen *Batrachochytrium dendrobatidis* (Bd) causes chytridiomycosis, an emerging infectious disease of amphibians [21]. Bd is widespread globally [22, 23], and is associated with worldwide amphibian population declines [19, 24]. Given IPCC climate projections, Bd is projected to increase its range, potentially placing additional amphibian populations at risk to Bd exposure [25].

The negative effects of Bd infection are more pronounced in post-metamorphic stages, often leading to death [26–29]. In larvae, Bd infection can cause host mortality in some species [27, 28]. However the infection is localized to keratinized larval mouthparts, [30, 31] often resulting in sublethal effects [26, 32, 33].

Bd is non-linearly sensitive to temperature with an optimal growth range in culture between 17° C and 25° C [34–36] and a temperature-dependent generation time of 4 to 10 days [37], both physiological characteristics of which can differ between strains [38]. The upper thermal limit for Bd growth in culture is between 25° C and 28° C, with Bd mortality occurring above 30° C [21, 34]. Bd has been shown to be reliably cleared from multiple amphibian species by extended exposure to 30° C [39]. Its lower thermal limit is below 4° C [34]. Additionally, life history strategies of the pathogen can be altered by environmental temperature, where colder temperatures can cause Bd zoosporangia to develop and mature more slowly [40], but produce more and longer-lived zoospores overall [37, 41]. Because physiologies of both the host and pathogen are strongly influenced by environmental temperature, climate change has been used to explain several major Bd outbreaks and amphibian population declines, [reviewed in 15, 42]. Yet, the host and pathogen are not expected to share a uniform response to a given temperature [42–44], and thermal responses measured in constant-temperature artificial environments might not reflect organism responses in more realistic variable-temperature environments. Providing evidence of the lack of a uniform response between Bd and amphibians to temperature shifts, Rohr and Raffel [35] found a strong correlation between elevated month-to-month temperature variability and Bd-associated amphibian population declines of *Atelopus* spp. across Central and South America. Further support of the relationship between chytridiomycosis and temperature variation has been provided by laboratory studies. In one study, Cuban treefrogs (*Osteopilus septentrionalis*) displayed reduced resistance to Bd infection when exposed to random daily temperature fluctuations or when exposed to a temperature decrease after acclimation to a warmer temperature [36]. Similar results were obtained in newts (*Notophthalmus viridescens*) exposed to Bd, except both decreases and increases in temperature were associated with elevated Bd abundance relative to abundances at constant temperatures [12].

**Fig 1:**
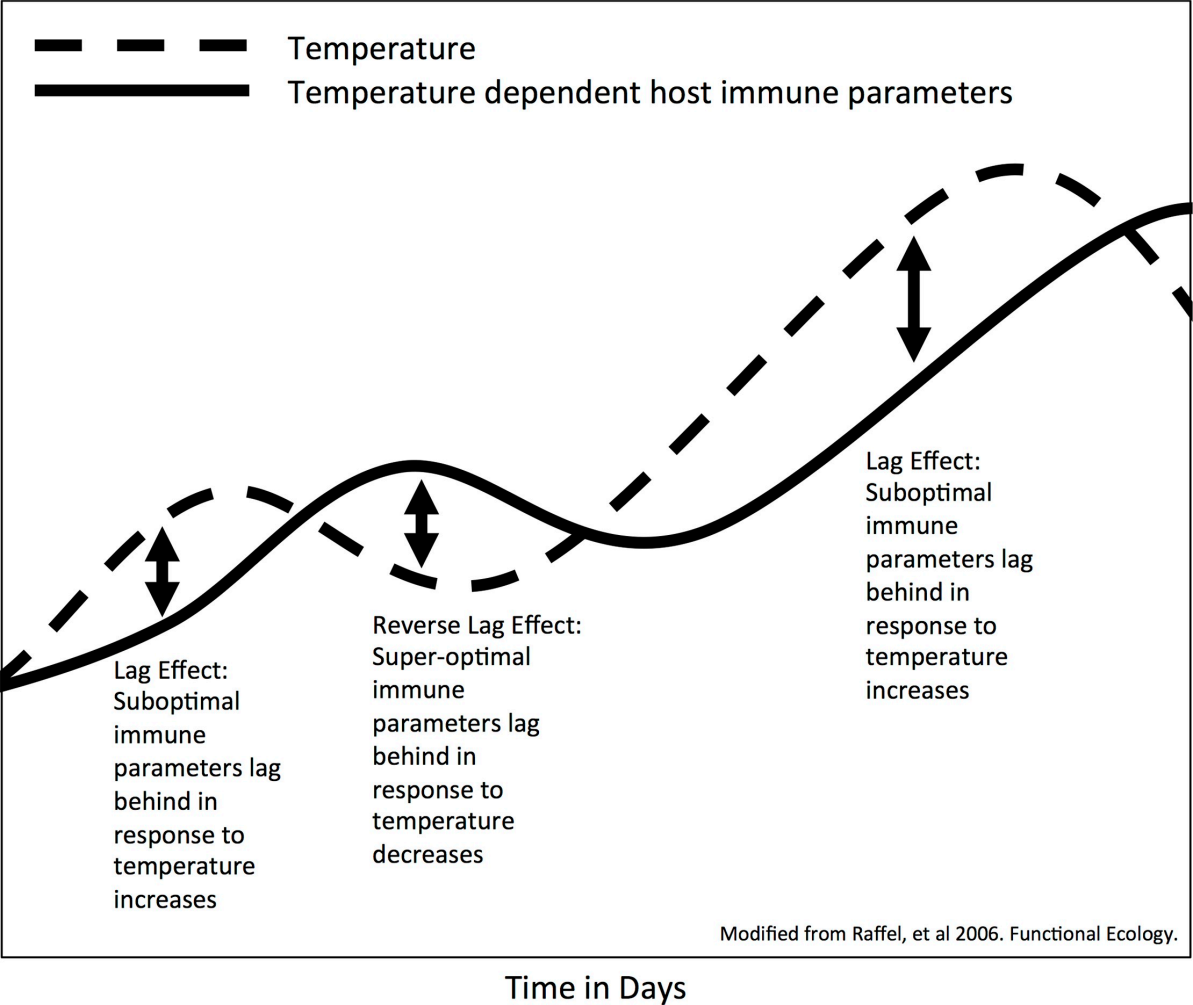
Hypothesized lag effect. Hypothesized lag effect showing the relationship between fluctuating temperatures (over days to weeks) and the optimal levels of a hypothetical temperature-dependent host immune parameter. The immune parameter follows and lags behind temperature changes–resulting in periods of a compromised immune status after a temperature increase, and resulting in an over-active (or unnecessarily costly) immune status after a temperature decrease. Modified from Raffel et al. (2006).

The potential for temperature variability to increase disease severity in amphibians was first postulated by Raffel et al. [45] and has subsequently been referred to as the “climate variability hypothesis” [35]. This hypothesis posits that parasites acclimate to the new temperature more rapidly than their hosts, leading to lags in host acclimation following a temperature shift that could make hosts more susceptible to infection [36]. However, Raffel, Rohr [45] also pointed out potential complexities in acclimation of the ectotherm immune system that may lead to alternative predictions. According to the “lag effect” hypothesis [35, 45], changes in levels of temperature-dependent immune parameters may simply lag behind environmental temperature shifts (Fig 1) because it takes time to produce necessary, or remove unnecessary, immune cells from the host. Thus, the “lag effect” hypothesis predicts the opposite effect from the “climate variability hypothesis” following a temperature decrease, at least on a short timescale. These mechanistic hypotheses are not mutually exclusive, and it is unclear which effects may be more important for a given host-parasite combination.

We tested the general prediction that an amphibian shifted to a new temperature before Bd exposure would respond to infection differently than a host already acclimated to the exposure temperature. We postulated that the direction of the effect would depend upon the direction of the temperature shift, in accordance with the “lag effect” hypothesis [35, 45]. Given the differences in size between the host and the pathogen, and associated physiological process rate differences, we assumed Bd would physiologically respond to the temperature shift faster than the host, such that an idealized host-immune response to Bd exposure would temporarily lag behind the temperature shift. Thus, we predicted that a temperature shift from cold-to-warm would result in an *increase* in susceptibility to Bd exposure, whereas a temperature shift from warm-to-cold would result in a *decrease* in susceptibility to Bd exposure.

## Materials and methods

In a laboratory study, we examined how temperature shifts may alter larval amphibian infection dynamics. We selected two species of amphibian hosts, the northern red legged frog (*Rana aurora*) and the western toad (*Anaxyrus boreas*) as adults of both species have been observed in the field with Bd infections [46–48] and both species are susceptible to chytridiomycosis [26, 32]. To ensure that the animals used in our experiment were not previously infected with Bd, amphibians were collected as eggs from natural oviposition sites where Bd is not known to be endemic. Red legged frog eggs were collected from a permanent pond located near Florence, Oregon, USA (Lincoln County, elevation 12 m; latitude/longitude: 44.088/-124.123) in the Oregon Coast Range on 11-Feb-2012. Western toad eggs were collected from a lake near Sisters, Oregon (Deschutes County, elevation 2,000 m; latitude/longitude: 44.009/-121.643) in the Cascade Range on 9-Jul-2011. Immediately after collection, eggs were transported to a laboratory at Oregon State University where they were maintained at 14° C, under a 12–12 photoperiod in 40-liter aquaria filled with dechlorinated water. Upon hatching, larvae were maintained in aquaria and fed *ad libitum* a mixture of Tetramin fish food and ground alfalfa pellets (1:3 ratio by volume). Water was changed every seven days. The 40-day trials for each species were not run concurrently, but identical protocols were used for both species and both trials consisted of individuals of identical larval stage (Gosner stage 26).

### Acclimation period

Independent trials for each host species began with a 20-day acclimation period with 80 individual larvae (Gosner stage 26) randomly selected and individually placed into 80 plastic 500-mL containers where they were housed for the duration of the acclimation period and experiment. Each container was filled with 14° C dechlorinated water and covered with a lid to help maintain water temperature and limit evaporation. Each container had 2-mm diameter holes drilled between the water line and the lid to allow air circulation into the container. Pairs of containers were then placed within 40 individual temperature-controlled chambers (to ensure independent replication of the temperature treatments) that were set at 15° C to avoid heat-shocking the larvae. Each temperature-controlled chamber was independently controlled via its own thermostat and the interior measured approximately 37-cm deep x 21-cm wide x 13-cm in height. Half of the 40 temperature-controlled chambers were then randomly selected to begin the acclimation period at 20° C (warm treatment) and the other half were kept at 15° C (cold treatment). The placement of temperature chambers within the laboratory was randomized, as was the placement of 500-mL containers within each temperature chamber.

### Temperature shifts

On day 20 of the experiment, half of the temperature chambers in each of the two acclimation temperatures (15° C and 20° C) were randomly selected to undergo a temperature shift, either from 20° to 15° C or from 15° C to 20° C. The other half of the temperature chambers underwent no shift in temperature. Thus, each of the temperature chambers was subjected to one of four temperature treatments: a constant 15° C (cold) throughout the experiment; a constant 20° C (warm) throughout the experiment; a temperature shift from 15° C to 20° C (cold-to-warm); or a temperature shift from 20° C to 15° C (warm-to-cold).

### Bd exposure

On day 24, four days after the water temperature shift for chambers in the two temperature shift treatments, individual larva underwent their exposure treatment. One of the two 500-mL containers within each of the 40 temperature-controlled chambers was randomly selected to undergo a Bd-exposure treatment and the other was selected as a control. Thus for each species, the 40 larvae in the Bd-exposure treatment were exposed to a single inoculate of Bd strain JEL 274, which was grown in pure culture on 1% tryptone agar in 10-cm diameter Petri dishes. The Petri dishes were inoculated with liquid culture 10 days before the start of the experiment and incubated at 15° C. To harvest the zoospores, 10 plates were flushed with 15 mL of 15° C dechlorinated water and remained undisturbed for 10 minutes. The plates were scraped with a rubber spatula to release the zoospores and sporangia adhering to the agar. The inoculum from each plate was then pooled in a beaker and the number of moving zoospores was determined using a hemocytometer and then diluted to 10,000 zoospores/mL. Individuals in the Bd-exposed treatments were exposed to 10 mL of inoculum transferred into the 500-mL container housing an individual larva. The 40 individuals in the control treatment were exposed to 10 mL of sham inoculum lacking the Bd culture (made from 1% tryptone sterile agar plates following the same methods), similarly transferred into the 500-mL container housing each larva.

During the 40-d trial larvae were monitored daily. Water for each 500-mL container within the temperature chambers was changed every 12 days and consisted of dechlorinated water of the same temperature (15° C and 20° C). As scheduled, day 24 of the experiment consisted of a water change that occurred prior to the exposure-treatment later that day. Individuals that survived until the end of the trial (i.e., day 40) were euthanized in a 2% solution of MS-222, and then preserved in 95% ethanol. Individuals that reached metamorphosis (Gosner stage 42: emergence of forelimbs) were euthanized and preserved as previously described but not included in the statistical analysis.

Only individuals previously trained in ethical animal care conducted data collection and animal monitoring and all efforts were made to minimize suffering. Criteria for euthanasia included display of overt signs of morbidity and individuals were checked daily. Any animals appearing to show any signs of distress were immediately euthanized in MS-222 according to institutional animal care protocol. During the 40-d experiment, no larval stage tadpoles were euthanized; all euthanized individuals in this study were at or near metamorphic climax (S1 Table).

### Determining infection status

We used quantitative polymerase chain reaction (qPCR) to determine infection status and quantify Bd-infection intensity of all individuals in the Bd-exposure treatments. Additionally, we investigated Bd-infection status in eight randomly selected control individuals per species. To sample the individuals for Bd, we extracted whole mouthparts of the larvae using sterile dissection scissors. We bead-beated the mouthparts and conducted qPCR using an ABI PRISM 7500 sequencer (Applied Biosystems) according to the methods of Boyle *et al*. [49] except that we used 60 μL of Prepman Ultra (Applied Biosystems, Carlsbad, California, USA), instead of the 40 μL in the DNA extraction. All samples were run in triplicate and averaged.

### Statistical analyses

Each temperature-controlled chamber was subjected to one of four temperature regimes consisting of a Bd-exposure temperature combined with a temperature shift status (constant cold, constant warm, shifted to cold, and shifted to warm). Further, the pairs of containers within each temperature-controlled chamber were subjected to one of two exposure treatments (Bd exposed and Bd unexposed).

Survival was compared between temperature treatments for western toad larvae with a Cox proportional hazards model using TIBCO Spotfire S+ version 8.1. The model consisted of the main effects of the temperature treatment, temperature shift status (constant versus shifted), and an interaction between the two variables. Due to losses of western toad larvae prior to Bd exposure, we lacked the power to statistically compare survival in western toad larvae between the Bd exposure treatments, whereas we had sufficient power to compare survival between temperature treatments for this species.

We were surprised by the losses observed in the western toad larvae during the acclimation period (prior to day 20) and in particular those individuals that died while experiencing the cold temperature (15° C) treatment. Both temperature extremes selected for this study are within the pre-metamorphic thermal tolerances of both amphibian species [50–52]. Additionally, this temperature range is environmentally relevant for breeding ponds for these species [53] near where the western toad eggs were collected and this temperature range has been used in previous laboratory studies with these species [53].

Bd infection abundance (Bd genomic equivalents) among temperature treatments and between host species was analyzed using R version 3.11. We used a zero-inflated negative-binomial generalized linear model (function ‘zeroinf’ in package ‘pscl’) as described by Raffel, Michel [54]. Our full model investigated the effects of all of the explanatory variables including host species, exposure temperature, temperature shift status, and all two- and three-way interactions on Bd abundance. Interpretation of this analysis required further reduced models to investigate the effect of exposure temperature and temperature shift for each species (species model) and the effect of temperature shift for each Bd-exposure temperature and host species combination (Bd-exposure temperature model).

## Results

### Survival

Survival differences were not detected between exposure temperatures (Cox, Z = -1.099, *p* = 0.27) or temperature shift status (Cox, Z = -0.277, *p* = 0.78) in Bd-exposed western toad larvae. Although some western toad individuals died or metamorphosed before the end of the experiment and thus were not tested for Bd at the end of the experiment, the statistical model for Bd abundance on western toads that we describe below was not significantly improved by adding pre/post-metamorphic state or sampling date as covariates (χ^2^_1_ = 3.33, *p* > 0.05). Therefore, we omitted both covariates from the final Bd abundance models for western toads. We were unable to detect survival differences in red legged frog larvae, as only one individual larva died after application of the exposure treatment.

### Infection abundance

We detected a host species-by-temperature shift interaction (χ^2^_1_ = 3.83, *p* = 0.050; S2 Table) and a Bd-exposure temperature-by-temperature shift interaction (χ^2^_1_ = 7.50, *p* = 0.006; S2 Table). We investigated these interactions with reduced models to investigate effects on Bd abundance at the levels of species and exposure temperature.

**Fig 2:**
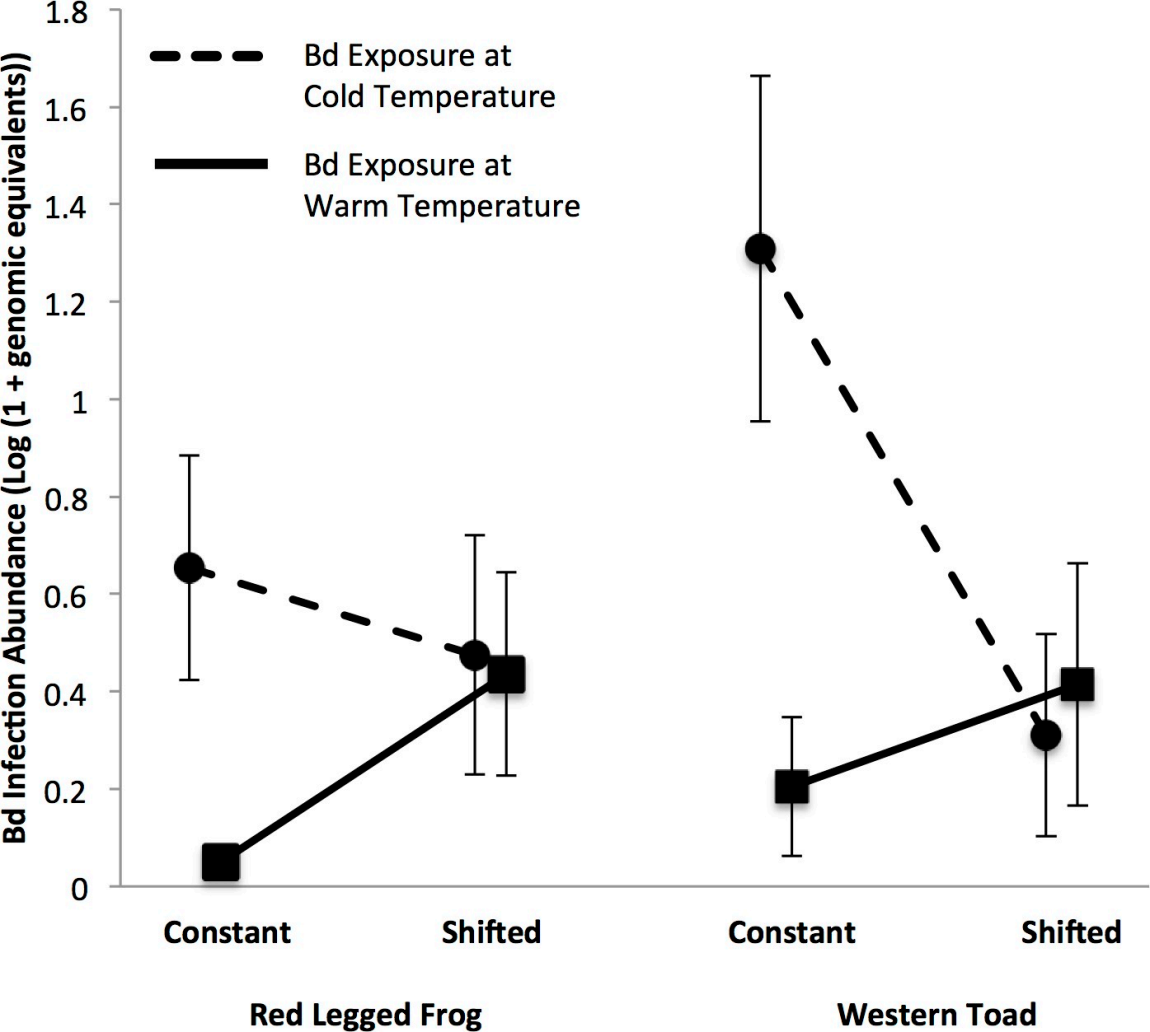
Mean *Batrachochytrium dendrobatidis* (Bd) infection abundance. Mean *Batrachochytrium dendrobatidis* (Bd) infection abundance (± SE) measured at death, or at euthanasia 16-days after Bd exposure, in both western toad (*Anaxyrus boreas*) larvae and red legged frog (*Rana aurora*) larvae from Oregon, USA, and between the two temperatures at the time of Bd-exposure (cold [15° C] versus warm [20° C]) and between larvae having experienced either a constant or shifted temperature. Bd infection abundance is quantified as the log (1 + Bd genomic equivalents) per excised larval mouthparts of all individuals exposed to the pathogen.

Red legged frog larvae had higher Bd abundance when they were exposed to Bd at 15° C when compared to 20° C (χ^2^_1_ = 3.88, *p* = 0.049; Fig 2). The main effect of temperature shift was not significant in the reduced species model analysis (χ^2^_1_ = 3.50, *p* = 0.061), but there was a significant effect of temperature shift for individuals exposed at 20° C in the reduced model of Bd-exposure (χ^2^_1_ = 5.7, *p* = 0.017), with individuals shifted from 15° C to 20° C having higher Bd abundance than red legged frog larvae experiencing constant 20° C (Fig 2). In contrast, there was no evidence that a temperature shift influenced Bd infection when red legged frog larvae were exposed to Bd at 15° C (χ^2^_1_ = 0.6, *p* = 0.4; Fig 2). There was no statistically significant interaction between exposure temperature and temperature shift for red legged frog larvae (χ^2^_1_ = 2.4, *p* = 0.13).

We detected an interactive effect of exposure temperature and temperature shift on Bd abundance in western toad larvae (χ^2^_1_ = 5.2, *p* = 0.023). This was driven by elevated Bd abundance in individuals under the constant 15° C temperature when compared to individuals that experienced a temperature shift from 20° to 15° C, but no evidence of an effect of shifting temperature from 15° C to 20° C (Fig 2). There were no main effects of exposure temperature (χ^2^_1_ = 0.50, *p* = 0.5) or temperature shift (χ^2^_1_ < 0.01, *p* = 0.9) on Bd abundance in western toad larvae. Further, when investigating the exposure temperatures individually in the reduced model of Bd-exposure, there was no evidence that a temperature shift influenced Bd infection in western toad larvae after exposure to Bd at 15° C (χ^2^_1_ = 3.4, *p* = 0.066) or 20° C (χ^2^_1_ = 2.5, *p* = 0.11).

We failed to find evidence that the two host species differed in responses to the pathogen (χ^2^_1_ = 2.57, *p* = 0.109), leading us to conclude that general patterns for both species were similar (Fig 2).

## Discussion

Numerous climate change models predict increases in annual or seasonal mean temperatures in many locations [55]. These models often also predict elevated chances of extreme weather events [3, 6]. Temperature shifts that may be associated with the onsets and conclusions of these weather events have the potential to alter species interactions–including host-pathogen interactions [10, 11, 35].

Our results suggest that Bd infection dynamics in larval amphibians can be affected by a shift in water temperature before host exposure to the pathogen, and that the direction of temperature shift affects the outcome of Bd exposure. Importantly, we detected the effects of temperature shifts despite the host having a four-day head start on acclimating to the Bd exposure temperature relative to the pathogen. This suggests that we may underestimate the strength of these effects and that their magnitudes may have been larger if the host and pathogen experienced the shifts concurrently, which probably would be common in the field.

Amphibian species do not all respond similarly to a given Bd exposure. Species-level differences in host tolerance to Bd infections have been well documented under controlled laboratory conditions [29, 56]. Under natural conditions, pathogen tolerance within a species may be affected by biotic factors such as inter- and intra-specific interactions, proximity to metamorphosis, or life stage [27, 31, 57, 58] or abiotic factors such as temperature, season, or resource availability [54, 59]. For some susceptible host species, temperature-shift induced changes in Bd abundance may alter the outcome of infection by either pushing Bd abundance over or under a tolerance threshold.

Whereas results of similar studies investigating post-metamorphic red-spotted newts and Cuban treefrogs support the “climate variability hypothesis.” our results for the larval life-stage of western toads and red legged frogs were consistent with predictions of the “lag effect” hypothesis [35, 45], and were generally consistent with previous studies showing that a shift in temperature influences Bd infection in postmetamorphic amphibians [12, 36]. In particular, our finding in larval red legged frog of decreased resistance to infection (increased Bd abundance) following a temperature shift from cold to warm (relative to warm-acclimated individuals) mirrored a laboratory study of post-metamorphic red-spotted newts (*Notophthalmus viridescens*), where juvenile newts exhibited decreased Bd resistance following a shift from 15° C to 25° C [12]. These findings of fluctuating temperature effects on Bd infection across four anuran taxonomic groups and life-stages suggest that effects of temperature shifts and Bd-related chytridiomycosis susceptibility might be widespread within amphibians. However, our finding in larval western toads of increased resistance to Bd infection (decreased Bd abundance) following a temperature shift from warm to cold (relative to cold-acclimated individuals) was opposite the pattern observed in red-spotted newts and Cuban treefrogs [12, 36]. These contrasting results suggests that there are important among-taxa, among-stage, or among-experiment differences driving the effects of temperature fluctuation on Bd infection. Whereas our results in pre-metamorphic life-stage of western toads and red legged frogs are consistent with the “lag effect” hypothesis, results of similar studies investigating post-metamorphic red-spotted newts and Cuban treefrogs support the “climate variability hypothesis.”

Higher Bd abundances were observed for both host species under the constant cold temperature treatment compared to the constant warm temperature treatment. These results are consistent with previous experiments that showed increased Bd abundance [12] and Bd-induced mortality [12, 60] were associated with lower temperatures. This is despite Bd growing best in culture at about 23° C, which is much closer to the warm than cold temperature treatments in this experiment [34, 37].

Elevated Bd abundances under the constant cold temperature treatment compared to the constant warm temperature treatment may be because the larval immune response to Bd infection increases with increasing temperatures at a faster rate than the infectivity or growth rate of Bd [36], or alternatively because of the differences between the growth rate of Bd in culture compared to the growth rate on host tissue [61]. Our results provide further evidence to suggest patterns of Bd growth in culture differ from patterns of Bd growth on a host and that it is important to assess the host-parasite interaction when predicting effects of climate and climate change on disease risk.

Alternatively, differences in Bd abundance between the two constant temperature treatments may be due to temperature effects on the pathogen rather than the host [37, 40]. The Bd was cultured at 15° C; it is possible that the temperature shift experienced by the pathogen in the warm exposure treatment caused the depressed Bd abundances observed in both host species compared to the elevated Bd abundance in the cold exposure temperature treatment. A decrease in temperature may cause an increase in the number of Bd zoospores released from zoosporangia [37, 41], however the effect of a similar increase in temperature on Bd physiology is unclear.

The “thermal mismatch hypothesis” suggests ectothermic hosts should on average be more susceptible to infection at temperatures that most greatly differ from the temperature at which they are most well adapted. This notion has been used to help explain the variation in species responses to Bd across space and time [62]. Red legged frogs are generally more common at lower elevations than western toads and thus might be more warm-adapted [63]. If so, the thermal mismatch hypothesis would predict that they would have more Bd than western toads at cooler temperatures. Nevertheless, there was no striking difference in Bd growth on the two species across temperatures (Fig 2). These patterns could simply be due to a weak difference between the relative temperature adaptations or preferences of the two host species given that they were collected from nearby locations or unique features of the species combination that generated patterns inconsistent with the broader patterns of the thermal mismatch hypothesis.

In conclusion, our results provide additional evidence for climate variability affecting Bd infection in amphibians but suggest important among-taxa, life-stage, or experiment differences in the directionality of these effects. Our study highlights the complexity that temperature plays in determining the outcome of Bd-amphibian interactions and the role that fluctuating temperature may play in altering these interactions. Furthermore, this study increases the diversity of amphibian species and stages that have been shown to exhibit thermal acclimation effects on disease, and suggests that fluctuating-temperature effects on amphibian infection might be widespread.

## Supporting information

S1 Table Summary survival information. Proximate causes of death for individual amphibians of both host species within each treatment and across the time periods of the 40-d study.
(DOCX)

S2 Table Full model investigating *Batrachochytrium dendrobatidis* (Bd) abundance. Full model investigating the effects of host species, exposure temperature, temperature shift status, and all two- and three-way interactions on *Batrachochytrium dendrobatidis* abundance on red legged frog larvae (*Rana aurora*) and western toad larvae (*Anaxyrus boreas*) from Oregon, USA.
(DOCX)

S1 Data Metadata and dataset collected in the study. (CSV)
